# Inflammatory state exists in familial amyloid polyneuropathy that may be triggered by mutated transthyretin

**DOI:** 10.1038/s41598-017-01775-4

**Published:** 2017-05-08

**Authors:** Genki Suenaga, Tokunori Ikeda, Teruaki Masuda, Hiroaki Motokawa, Taro Yamashita, Kotaro Takamatsu, Yohei Misumi, Mitsuharu Ueda, Hirotaka Matsui, Satoru Senju, Yukio Ando

**Affiliations:** 1 Department of Neurology, Graduate School of Medical Sciences, Kumamoto University, Kumamoto, Kumamoto, Japan; 2 Department of Clinical Investigation, Kumamoto University, Kumamoto, Kumamoto, Japan; 3 Department of Clinical Laboratory, National Hospital Organization Kyushu Medical Center, Fukuoka, Fukuoka, Japan; 4 Department of Molecular Laboratory Medicine, Graduate School of Medical Sciences, Kumamoto University, Kumamoto, Kumamoto, Japan; 5 Department of Laboratory Medicine, Kumamoto University Hospital, Kumamoto, Kumamoto, Japan; 6 Department of Immunogenetics, Kumamoto University, Kumamoto, Kumamoto, Japan

## Abstract

The relationship between familial amyloid polyneuropathy (FAP), which is caused by mutated transthyretin (TTR), and inflammation has only recently been noted. To determine whether inflammation is present in FAP carriers and patients, serum interleukin (IL)−6 concentration in 57 healthy donors (HD), 21 FAP carriers, and 66 FAP patients was examined, with the relationship between IL-6 and TTR assessed in each group by multiple regression analysis and structural equation models (SEM). Compared with HD, IL-6 concentration was elevated in FAP carriers (*p* = 0.001, 95% CI 0.398–1.571) and patients (*p* = 0.002, 95% CI 0.362–1.521). Further, SEM indicated a positive relationship between IL-6 and TTR in FAP carriers (*p* = 0.010, 95% CI 0.019–0.140), but not in HD and FAP patients. In addition, we determined whether TTR induces production of pro-inflammatory cytokines *ex vivo*. HD-derived CD14 + monocytes and induced pluripotent stem cell-derived myeloid lineage cells from a HD and FAP patient dose-dependently produced IL-6 under mutated and aggregated TTR conditions, compared with wild-type TTR. In conclusion, FAP carriers and patients are in an inflammatory state, with the presence of mutated TTR being a trigger of inflammation, especially in FAP carriers.

## Introduction

Familial amyloid polyneuropathy (FAP) is a rare neurodegenerative disease showing autosomal dominant inheritance, which is caused by deposition of mutated transthyretin (TTR)-derived amyloid fibrils in several organs. Concentration of serum TTR in FAP patients is low compared with healthy subjects^1, 2^. Generally, bad nutrition and inflammation are blamed for decreased TTR concentration, yet advanced FAP patients are underweight^2–4^. Indeed, the relationship between FAP and inflammation is not obvious. In a FAP mouse model, administration of an interleukin (IL)−1 antagonist inhibited TTR deposition at the sciatic nerve^5^, while V122I mutated TTR affected expression of interleukin-6 (IL-6) in chondrocytes^6^. These results suggest that mutated TTR may affect pro-inflammatory cytokines. In this regard, although IL-1β and tumor necrosis factor (TNF)-α are expressed in the sural nerve of FAP patients, their expression is local and not systemic^7^. Here, to determine the presence of inflammation in FAP, we examined serum IL-6 concentration in FAP carriers and patients, and compared both groups to healthy donors (HD). We show that FAP carriers and patients are in an inflammatory state, and in particular, FAP carrier-derived TTR positively affects IL-6 concentration. Further, we confirmed this phenomenon using an *ex vitro* assay.

## Results

### Elevated serum IL-6 concentration in FAP carriers and patients

To determine the presence of inflammation, serum IL-6 and high-sensitivity C-reactive protein (hs-CRP) concentration were cross-sectionally analysed in HD (*n* = 57), as well as FAP carriers (*n* = 21) and patients (*n* = 66) (Supplementary Table S1). Concentration of IL-6 but not hs-CRP was higher in FAP carriers and patients than HD. However, because older age increases IL-6 levels^8^, we used multiple regression analysis adjusted by age to determine any differences in serum IL-6 concentration between the three groups (Table 1). We found that IL-6 was related to age (*p* = 0.046, 95% CI 0.000–0.030), with significantly elevated levels in FAP carriers (*p* = 0.001, 95% CI 0.398–1.571) and patients (*p* = 0.002, 95% CI 0.362–1.521) compared with HD. In contrast, there were no differences in hs-CRP concentration between the three groups (data not shown). To confirm this regression model, we used bootstrap testing (*n* = 2000) and found the same tendency (Supplementary Table S2). Next, we compared the V30M phenotype (*n* = 39) and other phenotypes (*n* = 27) in FAP patients (Supplementary Table S3). Our results show elevated IL-6 concentration in both groups, with no significant difference in between them.

**Table 1:** Multivariate regression model analysis.

Endogenous variable	Exogenous variable	Estimate	SE	t-value	*p*-Value	95% CI
Log (IL-6)	Intercept	−1.729	0.291	−5.94	<0.001	(−2.305, −1.154)
	age	0.015	0.008	2.01	0.046	(0.000, 0.030)
	HD (ref)					
	FAP carrier	0.985	0.297	3.32	0.001	(0.398, 1.571)
	FAP	0.942	0.293	3.21	0.002	(0.362, 1.521)

SE, standard error; CI, confidence interval; ref, reference.

### Structural equation models suggest TTR in FAP carriers but not HD drives IL-6 induction

**Figure 1:**
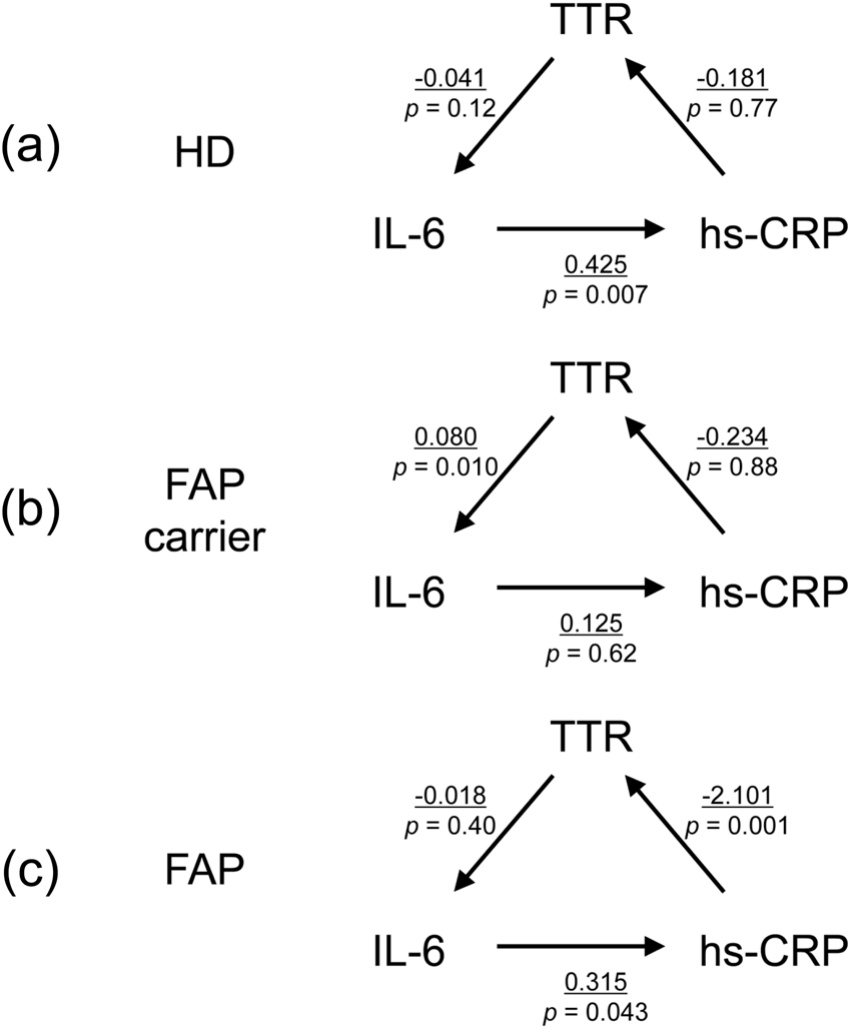
Path diagram showing multilevel linear model results as structural equation models without covariates. Estimates (underlined) and *p* values in each linear model are shown in healthy donors (HD) (**a**), familial amyloid polyneuropathy (FAP) carriers (**b**), and FAP patients (**c**). A detailed description can be found by reference to Supplementary Table S4. High-sensitivity C-reactive protein (Hs-CRP) and interleukin (IL)−6 were log-transformed to approximate a normal distribution.

Because FAP carriers and patients exhibit mutated TTR, with wild-type TTR at variance with HD, it is possible that mutated TTR affects IL-6 production. Therefore, to investigate the relationship between TTR and IL-6 in each group, we examined TTR concentration (Supplementary Tables S1 and S3) using structural equation models (SEM) without covariates (Fig. 1 and Supplementary Table S4). TTR significantly inhibited hs-CRP in FAP patients (*p* = 0.001, 95% CI −3.298–−0.904) but not HD or FAP carriers. Although IL-6 positively affected hs-CRP in HD (*p* = 0.007, 95% CI 0.117–0.732) and FAP patients (*p* = 0.043, 95% CI 0.009–0.621), this relationship was absent in FAP carriers (*p* = 0.62, 95% CI −0.374–0.625). Further, regarding the relationship between IL-6 and TTR, TTR in FAP carriers exerted a significantly positive effect (*p* = 0.010, 95% CI 0.019–0.140), which was not observed in HD (*p* = 0.12, 95% CI −0.091–0.100) or FAP patients (*p* = 0.40, 95% CI −0.058–0.023). Bootstrap testing (*n* = 2000) in SEM showed a similar tendency (Supplementary Table S5). Next, we examined differences in pathway parameters between HD and FAP carriers and patients (Table 2). The relationship between IL-6 and TTR was significantly different in FAP carriers compared with HD (*p* = 0.003, 95% CI −0.199–−0.042) and FAP patients (*p* = 0.009, 95% CI −0.170–−0.025), while there was no difference between HD and FAP patients (*p* = 0.49, 95% CI −0.088–0.042). Estimated differences between hs-CRP and IL-6 were not significant in HD compared with FAP carriers or patients, or between FAP carriers and patients. We examined SEM with covariates, in which the effect of age and sex were adjusted accordingly, as previously reported^8, 9^. This showed that the relationship between IL-6 and TTR manifested a similar tendency as in SEM without covariates analysis (Supplementary Figure 1, Supplementary Tables S6 and S7).

**Table 2:** Group differences in pathway parameters without covariates between HD and FAP carriers and patients.

Endogenous variable	Exogenous variable	Estimate	SE	Z	*p*-Value	95% CI
HD vs FAP carriers						
Log (hs-CRP)	Log (IL-6)	0.299	0.299	1.00	0.32	(−0.287, 0.886)
Log (IL-6)	TTR	−0.120	0.040	−2.99	0.003	(−0.199, −0.042)
TTR	Log (hs-CRP)	0.052	1.677	0.03	0.98	(−3.235, 3.340)
HD vs FAP patients						
Log (hs-CRP)	Log (IL-6)	0.109	0.221	0.49	0.62	(−0.325, 0.543)
Log (IL-6)	TTR	−0.023	0.033	−0.70	0.49	(−0.088, 0.042)
TTR	Log (hs-CRP)	1.920	0.866	2.22	0.027	(0.223, 3.616)
FAP carriers vs FAP patients						
Log (hs-CRP)	Log (IL-6)	0.190	0.299	0.64	0.53	(−0.396, 0.776)
Log (IL-6)	TTR	−0.097	0.037	−2.62	0.009	(−0.170, −0.025)
TTR	Log (hs-CRP)	−1.867	1.677	−1.11	0.27	(−5.153, 1.419)

HD, healthy donor; SE, standard error; CI, confidence interval.

### Mutated TTR increases IL-6 production in myeloid cells *ex vivo*

**Figure 2:**
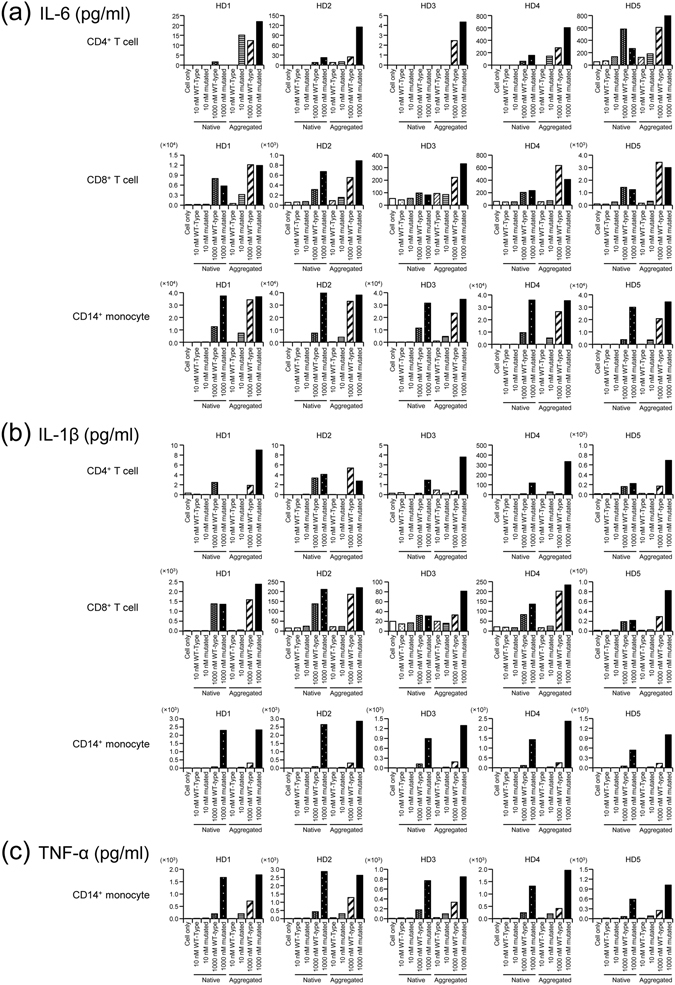
Mutated transthyretin induces pro-inflammatory cytokines in CD14 + monocytes. Five healthy donor (HD)-derived CD4 + and CD8 + T cells were stimulated by anti-CD3/anti-CD28 monoclonal antibodies (5 μg/ml) in the presence of 10 or 1000 nM native wild-type transthyretin (TTR), native V30M mutated TTR, wild-type-derived aggregated TTR, and V30M mutated-derived aggregated TTR for 5 days. HD-derived CD14 + monocytes were cultured with each type of TTR for 2 days. The Bio-Plex system was used to examine interleukin (IL)−1β (**a**) and IL-6 (**b**) concentration in culture supernatants of CD4 + T cells, CD8 + T cells, and CD14 + monocytes. Tumor necrosis factor (TNF)-α (**c**) concentration was also analysed in CD14 + monocytes.

As shown, IL-6 concentration was elevated in FAP carriers and patients compared with HD. In addition, the relationship between IL-6 and TTR in FAP carriers was different compared with HD and FAP patients. Because FAP carriers and patients both have mutated TTR, we determined if mutated TTR affects increased IL-6 concentration *ex vivo*. HD-derived CD4 + and CD8 + T cells, CD14 + monocytes, and induced pluripotent stem cell-derived myeloid lineage cells (iPS-MLs) originating from HD and FAP patients were cultured in the presence of native wild-type or V30M mutated TTR and aggregated TTR. Cytokines in culture supernatants were quantified using the Bio-Plex system. In native TTR culture conditions, IL-6 increased in CD14 + monocytes and iPS-MLs in the presence of V30M mutated TTR, compared with wild-type TTR, in a TTR-dose-dependent manner (Fig. 2a and Supplementary Figure 2). In contrast, although IL-6 concentration increased in CD4 + T cells and CD8 + T cells in a TTR-dose-dependent manner, there was no difference between V30M mutated and wild-type TTR (Fig. 2a). In aggregated TTR culture conditions, IL-6 concentration was elevated in the presence of V30M mutated TTR compared with wild-type TTR in CD14 + monocytes, iPS-MLs, and CD4 + T cells (but not CD8 + T cells) in a TTR-dose-dependent manner (Fig. 2a and Supplementary Figure 2). Further, the pro-inflammatory cytokines, IL-1β and TNF-α, and inhibitory cytokine, IL-10, increased in a TTR-dose-dependent manner in native and aggregated V30M mutated TTR conditions in CD14 + monocytes but not CD4 + T cells, CD8 + T cells, or iPS-MLs (Fig. 2b and c, Supplementary Figures 2 and 3a). Other cytokines, namely interferon (IFN)-γ and IL-15 (in all cell subsets), and IL-4, IL-7, and IL-12 (in CD14 + monocytes) were not dependent on the type nor dose of TTR (Supplementary Figures 2 and 3b–f).

## Discussion

We sought to determine if FAP carriers and patients are in an inflammatory state, and additionally, if presence of mutated TTR is involved in the inflammation. IL-6 is a pro-inflammatory cytokine that has been reported in chronic inflammatory diseases such as cancer, arteriosclerosis, and advancing age^8, 10–12^. Moreover, V122I mutated TTR affects IL-6 expression in chondrocytes^6^. Therefore, we focused on the pro-inflammatory cytokine, IL-6. We found increased serum concentration of IL-6 in FAP carriers and patients. Indeed, regardless of preclinical stage, FAP carriers were in an inflammatory state. Consistently, upregulation of inflammatory genes in peripheral blood cells from male FAP patients was recently reported^13^.

Next, we examined the relationship between TTR and IL-6 using SEM, which are multivariate regression models that can incorporate multiple regression equations^14^. For our purposes (i.e., to explore TTR and IL-6 involvement), hs-CRP (as an IL-6 related molecule) was added into the model. Although IL-6 positively regulated hs-CRP in HD, as previously reported^15^, this effect was weak in FAP patients and not confirmed in FAP carriers. In addition, differences in pathway parameters between HD and FAP carriers or patients were not significant. These results suggest that although IL-6 positively regulates hs-CRP in all groups, high IL-6 quantity in FAP carriers and patients induces uncertain correlation. For IL-6 and TTR involvement, only FAP carriers exerted a significant positive effect, with this pathway parameter also differentiated from the other two groups. These findings suggest that native mutated TTR may induce IL-6 in FAP carriers. Consequently, we determined whether IL-6 production was affected by mutated, mainly native TTR *ex vivo*. Accordingly, we confirmed that native V30M mutated TTR dose-dependently increased IL-6 concentration in CD14 + monocytes. In addition, instead of cell subsets from FAP patients, we used FAP patient-derived iPS-MLs (which function like macrophages^16^ and show a similar result in IL-6 concentration), as well as iPS-MLs derived from HD. However, although dose-dependency of native TTR was observed in IL-6 production in CD4 + and CD8 + T cells, there was no difference between V30M mutated and wild-type TTR. In aggregated TTR culture conditions, IL-6 production in CD14 + monocytes, iPS-MLs, and CD4 + T cells was elevated with V30M mutated TTR in comparison to wild-type TTR. In contrast, IL-6 concentration was higher in the presence of native and aggregated mutated TTR in CD14 + monocytes and iPS-MLs than CD4 + and CD8 + T cell conditions.

We selected the concentration of recombinant TTR in culture (1000 nM). Although culture conditions do not completely replicate *in vivo* conditions, as TTR circulates constantly in the blood^1, 2^, the dose of recombinant TTR used here is likely to influence IL-6 production in a physiologically realistic manner.

Wild-type TTR is usually present as a tetramer in healthy subjects. However, wild-type and mutated TTR heterotetramers are unstable in FAP carriers and patients, dissociating easily into wild-type and mutant monomers, with the latter being particularly susceptible to misfolding. Therefore, our *ex vivo* experiments of native TTR may reflect the *in vivo* occurrence of TTR monomers.

Megalin and the receptor for advanced glycation end products (RAGE) are known TTR receptors^17–19^. Although megalin expression in immune cells remains unknown, membranous expression of RAGE has been reported in human monocytes and T cells^20–22^. Moreover, intracellular RAGE expression is detected in human T cells following T cell receptor activation, and RAGE ligands may enhance RAGE expression via mechanisms such as endosomes^23, 24^. RAGE expression levels in monocytes were higher than in T cells, and the site of expression differed, suggesting that our observations may relate to differences in RAGE expression levels or locations in different cell subsets.

Wild-type TTR inhibits amyloid formation in Alzheimer’s disease^25^, and has an inhibitory effect on IL-1β production *in vitro* ^26^. However, using two types of TTR (wild-type and mutated TTR), increased IL-1β was found in FAP nerve and mouse models^5, 27^. Moreover, mutated TTR upregulates IL-6 expression in chondrocytes^6^. These results suggest that unlike wild-type TTR, mutated TTR easily initiates a pro-inflammatory state, and this phenomenon in FAP carriers may be a potential risk for FAP onset. Despite our result of elevated IL-6 concentration in FAP patients by multiple regression analysis, SEM did not show a significant and positive relationship between IL-6 and TTR. Considering our finding that aggregated TTR also produces IL-6 *ex vivo*, and the fact that FAP patients show amyloid fibril deposition originated from both wild-type and mutated TTR in several organs, it is possible that deposited amyloid, rather than mutated TTR, is the main inducer of pro-inflammatory cytokines such as IL-6. Consequently, the relationship might not be confirmed by SEM.

Our study has limitations with respect to the number of carriers and longitudinal data. Additionally, our FAP carriers and patients largely had a V30M phenotype. Thus, although our data shows that IL-6 concentration was also elevated in other phenotypes compared with HD, the number of these phenotypes was small and the IL-6 state in unexamined phenotypes is unknown. Therefore, whether elevated IL-6 in FAP carriers and patients is a common phenomenon for all phenotypes is not known. Besides, high IL-6 concentration in FAP may be attributed to causes other than existence of native mutated TTR. Further studies are needed to assess temporal changes in IL-6 concentration and the relationship between IL-6 and FAP onset in increased numbers of FAP carriers (including a wider variety of phenotypes). Nonetheless, we believe that mutated TTR may increase the risk of inflammation involving IL-6.

## Materials and Methods

### Blood samples

Serum samples were collected from 66 non-liver transplantation FAP patients (39 V30M, two V30M/V30M, one V30M/V50M, one F33V, one A36D, two G47R, two G47V, one T49I, two S50I, one S50R, one G53E, one L55P, one T59R, one T60A, one Q61K, one S77Y, one K80R, two E89K, two I107V, and three Y114C), 21 FAP carriers (14 V30M, three S50I, one I107V, and three Y114C), and 57 HD. FAP carrier diagnosis was determined by genetic analysis. FAP diagnosis was confirmed based on clinical phenomena, amyloid deposition in tissue, and genetic diagnosis. More clinical information on FAP carriers and patients is provided in Table 3 and 4. Samples were stored at −80 °C until the time of assay at Kumamoto University Hospital between 2010 and 2016. Written informed consent was obtained from all participants after the procedure had been fully explained. A cross-sectional study was performed using these serum samples, with further detailed information shown in Supplementary Tables S1 and S3. To prepare human CD4 + and CD8 + T cells, and CD14 + monocytes, blood samples from HD were collected at Kumamoto University Hospital. All experiments using human samples were performed in accordance with the Declaration of Helsinki and the approval of the Institutional Review Board of Kumamoto University (Permit Number: 1087).

**Table 3:** Characteristics of FAP Carriers.

Patient	Sex	Age	Mutation type
Carrier1	M	74	V30M
Carrier2	F	24	V30M
Carrier3	F	61	V30M
Carrier4	M	43	I107V
Carrier5	F	26	V30M
Carrier6	M	25	V30M
Carrier7	M	44	V30M
Carrier8	F	42	V30M
Carrier9	M	54	V30M
Carrier10	F	38	Y114C
Carrier11	M	30	Y114C
Carrier12	M	26	S50I
Carrier13	F	37	V30M
Carrier14	F	56	V30M
Carrier15	F	61	V30M
Carrier16	F	38	V30M
Carrier17	M	45	V30M
Carrier18	F	38	V30M
Carrier19	F	35	Y114C
Carrier20	M	41	S50I
Carrier21	M	32	S50I

M, male; F, female.

**Table 4:** Clinical Characteristics and initial symptoms in FAP patients at time of blood collection.

Patient	Sex	Age	Disease duration	Mutation type	Initial Symptoms	Main symptoms						Terapeutic agent
						Sensory disorder	Movement disorder	Autonomic dysfunction	Organ dysfunction			
									Heart	Eye (vitreus opacity)	Kidney	
FAP 1	M	72	100	V30M	Sensory disorder	+	+	+	+	−	−	Tafamidis
FAP 2	M	39	51	V30M/V50M	Autonomic dysfunction	+	+	+	−	−	−	Tafamidis
FAP 3	M	63	39	V30M	Sensory disorder, autonomic dysfunction	+	+	+	+	−	−	Tafamidis
FAP 4	M	71	27	V30M	Sensory disorder	+	+	NA	+	NA	NA	NA
FAP 5	M	74	11	V30M	Sensory disorder	+	−	−	+	NA	NA	−
FAP 6	M	68	42	V30M	Cardiac dysfunction	+	−	−	+	+	−	−
FAP 7	M	43	30	V30M	Sensory disorder	+	−	+	+	−	−	−
FAP 8	M	56	37	T60A	Autonomic dysfunction	+	+	+	+	−	−	Tafamidis
FAP 9	M	69	67	V30M	Sensory disorder	+	+	+	+	−	−	Tafamidis
FAP 10	M	75	23	K80R	Autonomic dysfunction	+	+	+	+	NA	NA	−
FAP 11	M	38	4	Y114C	Ocular dysfunction	−	−	−	−	+	−	−
FAP 12	M	59	13	T49I	Sensory disorder	+	+	+	+	−	−	−
FAP 13	F	39	58	V30M	Sensory disorder	+	+	+	+	−	+	−
FAP 14	F	34	9	V30M	Sensory disorder	+	−	+	+	+	−	−
FAP 15	F	46	9	G53E	Psychiatric symptom	NA	−	NA	NA	NA	+	−
FAP 16	F	69	8	G47V	Sensory disorder	+	−	+	+	NA	−	−
FAP 17	M	35	32	F33V	Ocular dysfunction	+	−	+	+	+	−	−
FAP 18	M	75	4	V30M	Ocular dysfunction	NA	NA	NA	NA	+	NA	NA
FAP 19	M	64	210	V30M	Cardiac dysfunction	+	+	+	+	−	−	NA
FAP 20	F	71	15	G47V	Movement disorder, autonomic dysfunction	+	+	+	+	−	−	−
FAP 21	F	59	48	A36D	Sensory disorder	+	−	−	+	−	−	−
FAP 22	M	69	77	V30M	Sensory disorder, cardiac dysfunction	+	+	+	+	+	−	−
FAP 23	M	61	53	V30M	Sensory disorder	+	+	+	+	−	−	−
FAP 24	M	72	113	V30M/V30M	Ocular dysfunction	+	+	+	−	+	−	Tafamidis
FAP 25	M	51	25	V30M	Sensory disorder	+	NA	NA	NA	NA	NA	NA
FAP 26	M	70	128	V30M/V30M	Ocular dysfunction	+	+	+	−	+	−	−
FAP 27	M	71	40	V30M	Sensory disorder	+	+	−	+	−	−	Tafamidis
FAP 28	M	69	76	I107V	Sensory disorder	+	+	+	+	−	−	−
FAP 29	M	57	27	I107V	Sensory disorder	+	+	NA	+	−	NA	−
FAP 30	M	66	49	V30M	Sensory disorder	+	−	−	−	−	−	Tafamidis
FAP 31	M	83	73	V30M	Sensory disorder	+	+	−	NA	NA	NA	−
FAP 32	M	57	4	S50I	Cardiac dysfunction	−	−	−	+	NA	−	Tafamidis
FAP 33	F	77	96	V30M	Sensory disorder	+	+	−	+	+	NA	−
FAP 34	M	64	96	V30M	Sensory disorder	+	+	+	−	−	+	−
FAP 35	F	51	23	E89K	Cardiac dysfunction	+	−	−	+	−	−	Tafamidis
FAP 36	F	49	32	E89K	Sensory disorder	+	−	−	+	−	−	−
FAP 37	F	67	96	V30M	Sensory disorder	+	+	+	+	NA	+	Tafamidis
FAP 38	M	66	46	V30M	Sensory disorder, movement disorder	+	+	+	+	NA	NA	−
FAP 39	F	35	15	V30M	Sensory disorder	+	−	−	−	−	−	−
FAP 40	F	41	13	Y114C	Sensory disorder	+	−	−	−	−	−	Tafamidis
FAP 41	M	79	18	Q61K	Cardiac dysfunction	+	+	+	+	NA	−	NA
FAP 42	M	73	71	V30M	Sensory disorder	+	+	+	+	−	−	Diflunisal
FAP 43	M	50	31	G47R	Sensory disorder, autonomic dysfunction	+	NA	+	NA	NA	NA	NA
FAP 44	M	65	55	V30M	Sensory disorder	+	+	+	+	−		−
FAP 45	M	71	36	V30M	Sensory disorder	+	+	+	+	−	+	Tafamidis
FAP 46	F	69	30	V30M	Autonomic dysfunction	+	−	+	−	−	+	−
FAP 47	M	69	49	V30M	Sensory disorder, movement disorder	+	+	+	+	+	−	−
FAP 48	M	31	16	L55P	Sensory disorder	+	+	+	+	−	−	−
FAP 49	F	46	51	G47R	Sensory disorder	+	N	+	+	+	+	NA
FAP 50	F	26	38	V30M	Sensory disorder	+	+	+	−	−	−	−
FAP 51	F	26	0	V30M	Sensory disorder	+	−	−	−	−	−	−
FAP 52	F	63	74	S50I	Cardiac dysfunction	+	+	+	+	−	−	−
FAP 53	M	70	N	V30M	NA	+	+	NA	+	NA	NA	−
FAP 54	M	47	5	S50R	Cardiac dysfunction	−	+	+	+	NA	NA	−
FAP 55	F	35	7	Y114C	Ocular dysfunction	+	−	−	−	+	−	−
FAP 56	M	72	26	V30M	Movement disorder	+	+	−	NA	NA	−	−
FAP 57	M	70	77	V30M	Movement disorder	+	+	+	+	−	−	Diflunisal
FAP 58	M	59	26	V30M	Autonomic dysfunction	+	+	+	+	+	−	−
FAP 59	M	64	127	V30M	Cardiac dysfunction	+	+	−	+	−	−	Tafamidis
FAP 60	M	59	43	T59R	Cardiac dysfunction	−	−	−	+	−	−	−
FAP 61	M	72	77	V30M	Sensory disorder	+	+	+	+	−	−	Tafamidis
FAP 62	M	86	35	V30M	Sensory disorder	+	+	−	+	NA	−	−
FAP 63	M	64	38	S77Y	Autonomic dysfunction	+	+	+	+	−	−	Tafamidis
FAP 64	M	77	43	V30M	Sensory disorder	+	+	+	+	+	−	−
FAP 65	M	73	NA	V30M	NA	NA	NA	NA	NA	NA	NA	NA
FAP 66	F	56	153	V30M	Movement disorder, autonomic dysfunction	+	+	+	+	−	+	NA

M, male; F, female; NA, not applicable.

### ELISA for serum samples

Stored serum samples were centrifuged for 15 minutes at 1,000× g before assays. Serum IL-6 levels were measured using human IL-6 Quantikine immunoassays (R&D Systems, Minneapolis, MN, USA). Serum TTR and hs-CRP concentration were determined at a central clinical laboratory in Kumamoto University Hospital.

### Generation of aggregated TTR

Recombinant human wild-type and V30M (mutated) TTR were purchased from Wako (Osaka, Japan). To generate aggregated wild-type or mutated TTR, each TTR was incubated as described previously^17, 28^. Briefly, each TTR (pH 4.0) was incubated at 37 °C for 24 h. To confirm production of aggregated TTR, thioflavin T (ThT)-based fluorimetric assays were performed. Aggregated wild-type or mutated TTR was diluted in 50 mM glycine/NaOH buffer (pH 9.5) containing 5 μM ThT, and ThT fluorescence intensity measured using a Hitachi F-2700 fluorescence spectrophotometer (Hitachi, Tokyo, Japan) (excitation wavelength, 442 nm; emission wavelength, 489 nm). Generation of aggregated TTR from wild-type and mutated TTR was confirmed (Supplementary Figure S4).

### Cell culture

Peripheral blood mononuclear cells (PBMCs) were isolated from the blood of HD (*n* = 5) using Ficoll-Paque (GE Healthcare, Buckinghamshire, UK). PBMCs were incubated on ice for 10 min with FcR blocking regent (Miltenyi Biotec, Bergish Gladbach, Germany). Next, CD14 + monocytes, and CD4 + and CD8 + T cells, were purified using the magnetic-activated cell sorting (MACS) cell sorting system (Miltenyi Biotec). Cell purity > 90% was confirmed using FACS Calibur (BD Biosciences, San Jose, CA, USA) (Supplementary Figure S5), and the following antibodies were used: fluorescein isothiocyanate (FITC)-conjugated anti-human CD4 (Clone: OKT4; Biolegend), phycoerythrin (PE)-conjugated anti-human CD8a (Clone: Hit8a; Biolegend), FITC-conjugated anti-human CD14 (Clone: HCD14; Biolegend). FITC-conjugated mouse IgG1κ (Clones: P3.6.2.8.1; eBioscience), FITC-conjugated mouse IgG2bκ (Clone: MPC-11; eBioscience), and PE-conjugated IgG1κ (Clone: MOPC-21; Biolegend) were used as isotype-matched controls. HD and FAP patient-derived human iPS-MLs were generated as described previously^16, 29^. iPS-MLs were maintained in culture with MEMα (Gibco, Osaka, Japan) supplemented with 10% foetal bovine serum (Sigma-Aldrich, St Louis, MO, USA), 100 μg/ml penicillin–streptomycin (Gibco, Carlsbad, CA, USA), 25 ng/ml human macrophage colony-stimulating factor (Prospec-Tany Technogene, Rehovot, Israel), and 50 ng/ml human granulocyte-macrophage colony-stimulating factor (Prospec-Tany Technogene) at 37 °C in 5% CO_2_, as previous described^30^. To investigate the effect of TTR in each cell type, CD4 + and CD8 + T cells (3 × 10^5^ cells/well), and CD14 + monocytes (3 × 10^5^ cells/well) were cultured in Opti-MEM (Gibco) with 10% heat-inactivated human plasma (individually matched to cells from the same individual) with 10 or 1000 nM native (wild-type or V30M mutated) or aggregated TTR (Wako). iPS-MLs (3 × 10^5^ cells/well) were also cultured in Opti-MEM supplemented with 10% foetal bovine serum using the same TTR conditions. CD4 + and CD8 + T cells were stimulated with 5 μg/ml plate-bound anti-CD3 and anti-CD28 monoclonal antibodies (BD Biosciences) for 5 days. CD14 + monocytes and iPS-MLs were cultured for 2 days, with each culture supernatant analysed using the Bio-Plex system (Bio-Rad Laboratories, Hercules, CA, USA).

### Bio-Plex cytokine array system

Culture supernatants were collected and centrifuged for 5 min at 15,000× g. Cytokine levels (CD14 + monocytes: IL-1β, IL-4, IL-6, IL-7, IL-10, IL-12, IL-15, IFN-γ, and TNF-α; CD4 + /CD8 + T cells and iPS-MLs: IL-1β, IL-6, IL-10, IL-15, and IFN-γ) in culture supernatants were measured using the Bio-Plex Pro Cytokine Assay kit (Bio-Rad).

### Statistical analysis

Because FAP is a rare neurodegenerative disease, sample size was determined with consideration of the number of outpatients to Kumamoto University hospital during the survey period. To ascertain a normal distribution of variables, Shapiro–Wilk’s test was performed. For univariate analysis, one-way analysis of variance or the Kruskal–Wallis test were used. Additionally, the pairwise *t* or Wilcoxon rank sum test with Bonferroni correction were used for continuous variables. For categorical variables, pairwise Fisher’s exact test with Bonferroni correction was performed. Multiple regression analysis was used to confirm differences in serum IL-6 concentration between groups. These analyses were performed using R version 3.3.1 (The R Foundation for Statistical Computing, Vienna, Austria). To investigate the significance and similarity of pathways between HD and FAP carriers and patients, SEM with observed measurements were used. The effect of age and sex were adjusted accordingly in the model^8, 9^. Some variables were log-transformed to approximate a normal distribution after visual investigation of a measurement’s distribution. STATA version 14.1 (Stata Corp., College Station, TX, USA) was used to fit the above models, with two-sided tests performed and the level of statistical significance set at *p* < 0.05.

## Electronic supplementary material

Supplementary Information
