# Supplementary material for: Inflammatory state exists in familial amyloid polyneuropathy that may be triggered by mutated transthyretin
Source: Sci Rep. 2017 May 8;7:1579. doi: 10.1038/s41598-017-01775-4 (PMC5431548; doi:10.1038/s41598-017-01775-4)
Supplement: Supplementary file 1 — Supplementary Information [file 41598_2017_1775_MOESM1_ESM.pdf]

Inflammatory state exists in familial amyloid polyneuropathy that may be triggered by mutated transthyretin

Genki Suenaga[1], Tokunori Ikeda[1, 2*], Teruaki Masuda[1], Hiroaki Motokawa[3], Taro Yamashita[1], Kotaro Takamatsu[1], Yohei Misumi[1], Mitsuharu Ueda[1], Hirotaka Matsui[4, 5], Satoru Senju[6], Yukio Ando[1*]

Author affiliations

[1]Department of Neurology, Graduate School of Medical Sciences, Kumamoto University, Kumamoto, Kumamoto, Japan
[2]Department of Clinical Investigation, Kumamoto University, Kumamoto, Kumamoto, Japan
[3]Department of Clinical Laboratory, National Hospital Organization Kyushu Medical Center, Fukuoka, Fukuoka, Japan
[4]Department of Molecular Laboratory Medicine, Graduate School of Medical Sciences, Kumamoto University, Kumamoto, Kumamoto, Japan
[5]Department of Laboratory Medicine, Kumamoto University Hospital, Kumamoto, Kumamoto, Japan
[6]Department of Immunogenetics, Kumamoto University, Kumamoto, Kumamoto, Japan

***Corresponding author**

Dr. Tokunori Ikeda, Department of Neurology and Department of Clinical Research Center, Graduate School of Medical Sciences, Kumamoto University, 1-1-1 Honjo, Kumamoto 860-8556, Japan; Tel: +81-96-373-5575; E-mail: ryousei@kumamoto-u.ac.jp

Dr. Yukio Ando, Department of Neurology, Graduate School of Medical Sciences, Kumamoto University, 1-1-1 Honjo, Kumamoto 860-8556, Japan; Tel: +81-96-373-5893; E-mail: andoy709@kumamoto-u.ac.jp

Supplementary Table S1. Comparison of characteristics in HD and FAP carriers and patients.

	HD	FAP Carrier	FAP	*p*-value		
	n = 57	n = 21	n = 66	**HD vs FAP carrier**	**HD vs FAP**	**FAP carrier vs FAP**
Age, median (y)	32	38	64	0.014	< 0.001	< 0.001
(IQR)	(24.00, 38.00)	(32.00, 45.00)	(50.25, 71.00)			
Male, *n* (%)	36 (63)	10 (48)	46 (70)	0.90	1.00	0.22
IL-6, median (pg/ml)	0.320	0.668	1.038	0.002	< 0.001	0.96
(IQR)	(0.150, 0.617)	(0.465, 1.871)	(0.646, 1.944)			
TTR, median (mg/dl)	27.31	22.43	18.94	0.001	< 0.001	0.41
(IQR)	(25.07, 29.51)	(20.92, 25.78)	(13.44, 25.29)			
hs-CRP, median (mg/dl)	0.023	0.027	0.036	1.00	0.41	1.00
(IQR)	(0.011, 0.067)	(0.011, 0.079)	(0.014, 0.065)			

HD, healthy donor; IQR, interquartile range.

Supplementary Table S2.

Results of multivariate regression model analysis (*n* = 2000 bootstrap replications).

Endogenous variable	Exogenous variable	Observed Estimate	Bootstrap SE	z	*p*-Value	95% CI
Log (IL-6)	Intercept	−1.729	0.329	−5.26	< 0.001	(−2.374, −1.085)
	age	0.015	0.009	1.80	0.07	(-0.001, 0.032)
	HD (ref)					
	FAP carrier	0.985	0.317	3.11	0.002	(0.365, 1.605)
	FAP	0.942	0.310	3.04	0.002	(0.335, 1.549)

SE, standard error; CI, confidence interval; ref, reference.

Supplementary Table S3. Comparison of characteristics in FAP patients with V30M and other mutations.

	V30M FAP	Other phenotype	*p*-value
	n = 39	n = 27	**V30M FAP vs Other**
Age, median (y)	69	57	0.009
(IQR)	(62.00, 72.00)	(46.00, 66.50)	
Male, *n* (%)	29 (74)	17 (63)	0.42
IL-6, median (pg/ml)	0.914	1.143	0.24
(IQR)	(0.585, 1.800)	(0.698, 2.781)	
TTR, median (mg/dl)	22.50	13.36	< 0.001
(IQR)	(18.36, 25.90)	(9.89, 17.78)	
hs-CRP, median (mg/dl)	0.028	0.044	0.38
(IQR)	(0.014, 0.050)	(0.018, 0.074)	

HD, healthy donor; IQR, interquartile range.

Other phenotypes: V30M/V30M, V30M/V50M, F33V, A36D, G47R, G47V, T49I, S50I, S50R, G53E, L55P, T59R, T60A, Q61K, S77Y, K80R, E89K, I107V, and Y114C.

Supplementary Table S4. Results of multilevel linear model analysis without covariates.

Endogenous variable	Exogenous variable	Estimate	SE	Z	*p*-Value	95% CI
**HD**						
Log (hs-CRP)	Intercept	−3.025	0.257	−11.79	< 0.001	(−3.528, −2.522)
	Log (IL-6)	0.425	0.157	2.70	0.007	(0.117, 0.732)
Log (IL-6)	Intercept	−0.076	0.746	−0.10	0.92	(−1.538, 1.387)
	TTR	−0.041	0.026	−1.57	0.12	(−0.091, 0.010)
TTR	Intercept	27.712	2.306	12.02	< 0.001	(23.192, 32.233)
	Log (hs-CRP)	-0.181	0.613	-0.30	0.77	(-1.383, 1.020)
**FAP carrier**						
Log (hs-CRP)	Intercept	−3.467	0.254	−13.64	< 0.001	(−3.965, −2.968)
	Log (IL-6)	0.125	0.255	0.49	0.62	(−0.374, 0.625)
Log (IL-6)	Intercept	−1.853	0.708	−2.62	0.009	(−3.241, −0.465)
	TTR	0.080	0.031	2.59	0.010	(0.019, 0.140)
TTR	Intercept	21.079	5.654	3.73	< 0.001	(9.998, 32.161)
	Log (hs-CRP)	-0.234	1.561	-0.15	0.88	(-3.294, 2.827)
**FAP**						
Log (hs-CRP)	Intercept	−3.213	0.168	−19.17	< 0.001	(−3.542, −2.885)
	Log (IL-6)	0.315	0.156	2.02	0.043	(0.009, 0.621)
Log (IL-6)	Intercept	0.476	0.423	1.12	0.26	(−0.353, 1.304)
	TTR	−0.018	0.021	−0.85	0.40	(−0.058, 0.023)
TTR	Intercept	12.635	2.111	5.99	< 0.001	(8.498, 16.772)
	Log (hs-CRP)	-2.101	0.611	-3.44	0.001	(-3.298, -0.904)

HD, healthy donor; SE, standard error; CI, confidence interval.

Supplementary Table S5.

Results of multilevel linear model analysis without covariates (*n* = 2000 bootstrap replications).

Endogenous variable	Exogenous variable	Observed Estimate	Bootstrap SE	Z	*p*-Value	95% CI
**HD**						
Log (hs-CRP)	Intercept	−3.025	0.315	−9.61	< 0.001	(−3.642, −2.408)
	Log (IL-6)	0.425	0.192	2.21	0.027	(0.047, 0.802)
Log (IL-6)	Intercept	−0.076	0.925	−0.08	0.94	(−1.888, 1.737)
	TTR	−0.041	0.032	−1.29	0.20	(−0.103, 0.021)
TTR	Intercept	27.712	2.745	10.09	< 0.001	(22.331, 33.093)
	Log (hs-CRP)	-0.181	0.700	-0.26	0.80	(-1.553, 1.190)
**FAP carrier**						
Log (hs-CRP)	Intercept	−3.467	0.301	−11.52	< 0.001	(−4.056, −2.877)
	Log (IL-6)	0.125	0.408	0.31	0.76	(−0.675, 0.925)
Log (IL-6)	Intercept	−1.853	0.815	−2.27	0.023	(−3.450, −0.256)
	TTR	0.080	0.037	2.14	0.033	(0.007, 0.153)
TTR	Intercept	21.079	8.393	2.51	0.012	(4.629, 37.530)
	Log (hs-CRP)	-0.234	2.324	-0.10	0.92	(-4.788, 4.321)
**FAP**						
Log (hs-CRP)	Intercept	−3.213	0.161	−19.97	< 0.001	(−3.528, −2.898)
	Log (IL-6)	0.315	0.176	1.79	0.07	(-0.030, 0.660)
Log (IL-6)	Intercept	0.476	0.388	1.23	0.22	(−0.285, 1.236)
	TTR	−0.018	0.017	−1.01	0.31	(−0.052, 0.017)
TTR	Intercept	12.635	1.896	6.67	< 0.001	(8.920, 16.350)
	Log (hs-CRP)	-2.101	0.607	-3.46	0.001	(-3.291, -0.911)

HD, healthy donor; SE, standard error; CI, confidence interval.

Supplementary Table S6. Results of multilevel linear model analysis with covariates.

Endogenous variable	Exogenous variable	Estimate	SE	Z	*p*-Value	95% CI
**HD**						
Log (hs-CRP)	Intercept	−4.267	0.662	−6.45	< 0.001	(−5.564, −2.970)
	Log (IL-6)	0.394	0.153	2.58	0.010	(0.095, 0.693)
	Age	0.037	0.019	1.97	0.049	(0.000, 0.074)
Log (IL-6)	Intercept	−0.479	0.837	−0.57	0.57	(−2.120, 1.161)
	TTR	−0.033	0.026	−1.24	0.21	(−0.085, 0.019)
	Age	0.006	0.017	0.34	0.74	(−0.027, 0.038)
TTR	Intercept	24.021	3.725	6.45	< 0.001	(16.721, 31.321)
	Log (hs-CRP)	-0.900	0.547	-1.64	0.10	(-1.972, 0.172)
	Age	0.093	0.082	1.14	0.25	(-0.067, 0.253)
	Sex	-5.153	1.484	-3.47	0.001	(-8.063, -2.244)
**FAP carrier**						
Log (hs-CRP)	Intercept	−4.435	2.450	−1.81	0.07	(−9.238, 0.368)
	Log (IL-6)	2.700	2.518	1.07	0.28	(−2.235, 7.635)
	Age	0.030	0.055	0.55	0.58	(−0.077, 0.137)
Log (IL-6)	Intercept	−5.113	2.228	−2.29	0.022	(−9.480, -0.745)
	TTR	0.278	0.108	2.57	0.010	(0.066, 0.490)
	Age	−0.026	0.031	−0.84	0.40	(−0.087, 0.035)
TTR	Intercept	-60.423	48.019	-1.26	0.21	(-154.538, 33.693)
	Log (hs-CRP)	-16.562	9.712	-1.71	0.09	(-35.596, 2.473)
	Age	0.863	0.496	1.74	0.08	(-0.110, 1.835)
	Sex	-21.119	11.509	-1.83	0.07	(−43.677, 1.439)
**FAP**						
Log (hs-CRP)	Intercept	−4.159	0.725	−5.74	< 0.001	(−5.579, −2.738)
	Log (IL-6)	0.251	0.160	1.57	0.12	(−0.062, 0.564)
	Age	0.016	0.012	1.34	0.18	(−0.007, 0.039)
Log (IL-6)	Intercept	−0.780	0.683	−1.14	0.25	(−2.119, 0.559)
	TTR	−0.022	0.019	−1.12	0.26	(−0.059, 0.016)
	Age	0.022	0.009	2.42	0.016	(0.004, 0.040)
TTR	Intercept	15.619	4.917	3.18	0.001	(5.981, 25.257)
	Log (hs-CRP)	-2.008	0.598	-3.36	0.001	(-3.181, -0.835)
	Age	-0.022	0.062	-0.35	0.73	(-0.143, 0.100)
	Sex	-4.606	1.912	-2.41	0.016	(-8.354, -0.858)

HD, healthy donors; SE, standard error; CI, confidence interval.

Supplementary Table S7.

Group differences in pathway parameters with covariates between HD and FAP carriers and patients.

Endogenous variable	Exogenous variable	Estimate	SE	Z	*p*-Value	95% CI
**HD vs FAP carrier**						
Log (IL-6)	TTR	−0.311	0.111	−2.79	0.005	(−0.529, −0.093)
	Age	0.032	0.035	0.90	0.37	(−0.037, 0.101)
TTR	Log (hs-CRP)	15.662	9.727	1.61	0.11	(-3.403, 34.726)
	Age	-0.770	0.503	-1.53	0.13	(-1.755, 0.216)
	Sex	15.966	11.605	1.38	0.17	(−6.779, 38.710)
Log (hs-CRP)	Log (IL-6)	-2.306	2.522	-0.91	0.36	(−7.249, 2.638)
	Age	0.007	0.058	0.12	0.91	(−0.107, 0.120)
**HD vs FAP**						
Log (IL-6)	TTR	-0.011	0.033	-0.35	0.73	(−0.075, 0.053)
	Age	−0.017	0.019	−0.87	0.38	(−0.054, 0.021)
TTR	Log (hs-CRP)	1.108	0.811	1.37	0.17	(-0.481, 2.698)
	Age	0.115	0.102	1.12	0.26	(-0.086, 0.316)
	Sex	-0.547	2.421	-0.23	0.82	(−5.292, 4.198)
Log (hs-CRP)	Log (IL-6)	0.143	0.221	0.65	0.52	(−0.290, 0.576)
	Age	0.021	0.022	0.95	0.34	(−0.022, 0.064)
**FAP carrier vs FAP**						
Log (IL-6)	TTR	-0.299	0.110	-2.73	0.006	(-0.514, -0.084)
	Age	0.048	0.032	1.49	0.14	(−0.015, 0.112)
TTR	Log (hs-CRP)	14.553	9.730	1.50	0.14	(-4.517, 33.624)
	Age	-0.884	0.500	-1.77	0.08	(-1.864, 0.096)
	Sex	16.513	11.667	1.42	0.16	(−6.354, 39.380)
Log (hs-CRP)	Log (IL-6)	-2.448	2.523	-0.97	0.33	(−7.393, 2.496)
	Age	-0.014	0.056	-0.25	0.80	(−0.124, 0.096)

HD, healthy donor; SE, standard error; CI, confidence interval.

Supplementary Figure 1

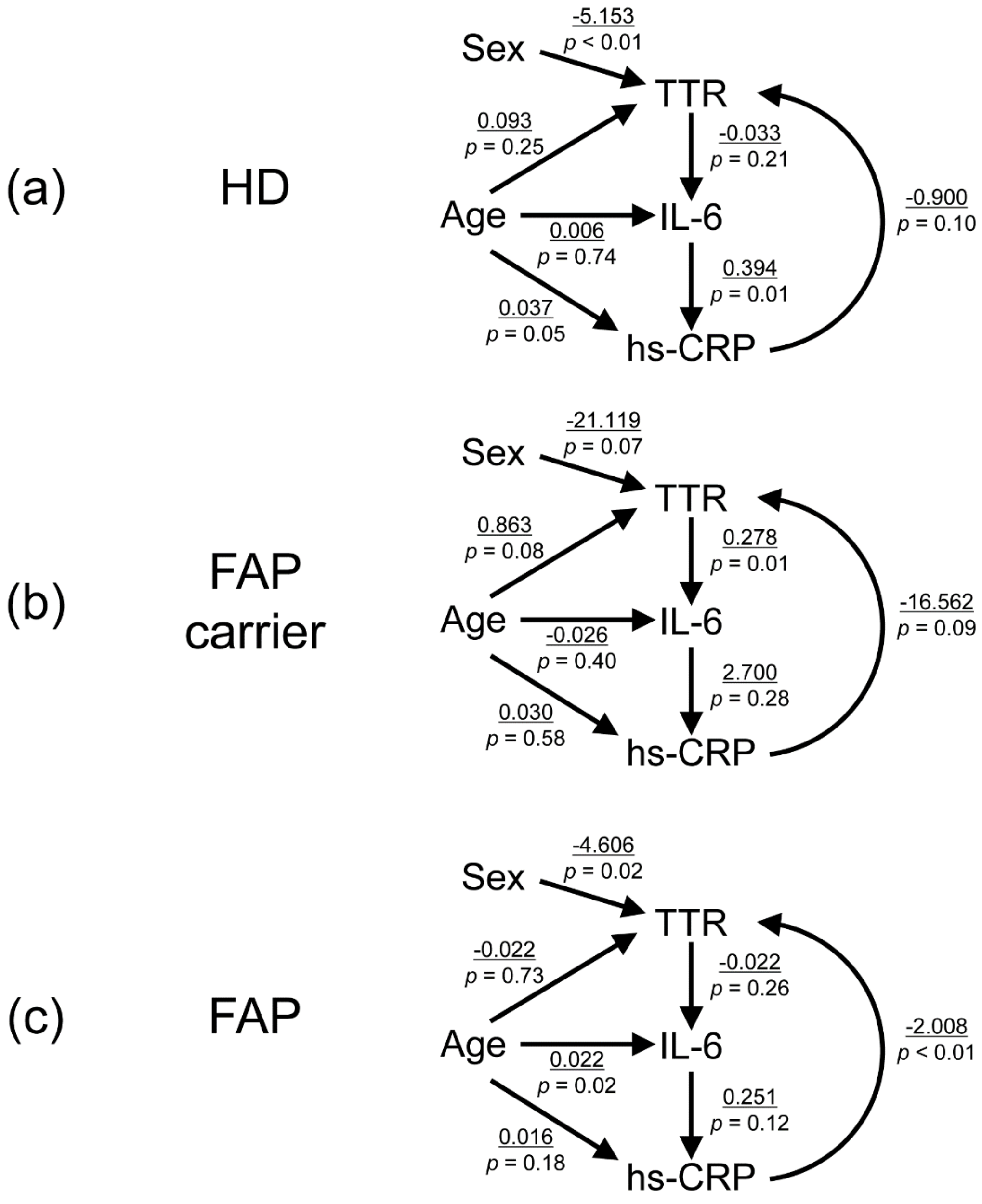

Supplementary Figure S1. Path diagram showing multilevel linear model results as structural equation models with covariates. Estimates (underlined) and *p* values in each linear model are shown in healthy donors (HD) (a), familial amyloid polyneuropathy (FAP) carriers (b), and FAP patients (c). A detailed description can be found by reference to Supplementary Table S6. High-sensitivity C-reactive protein (hs-CRP) and interleukin 6 (IL-6) were log-transformed to approximate a normal distribution.

Supplementary Figure 2

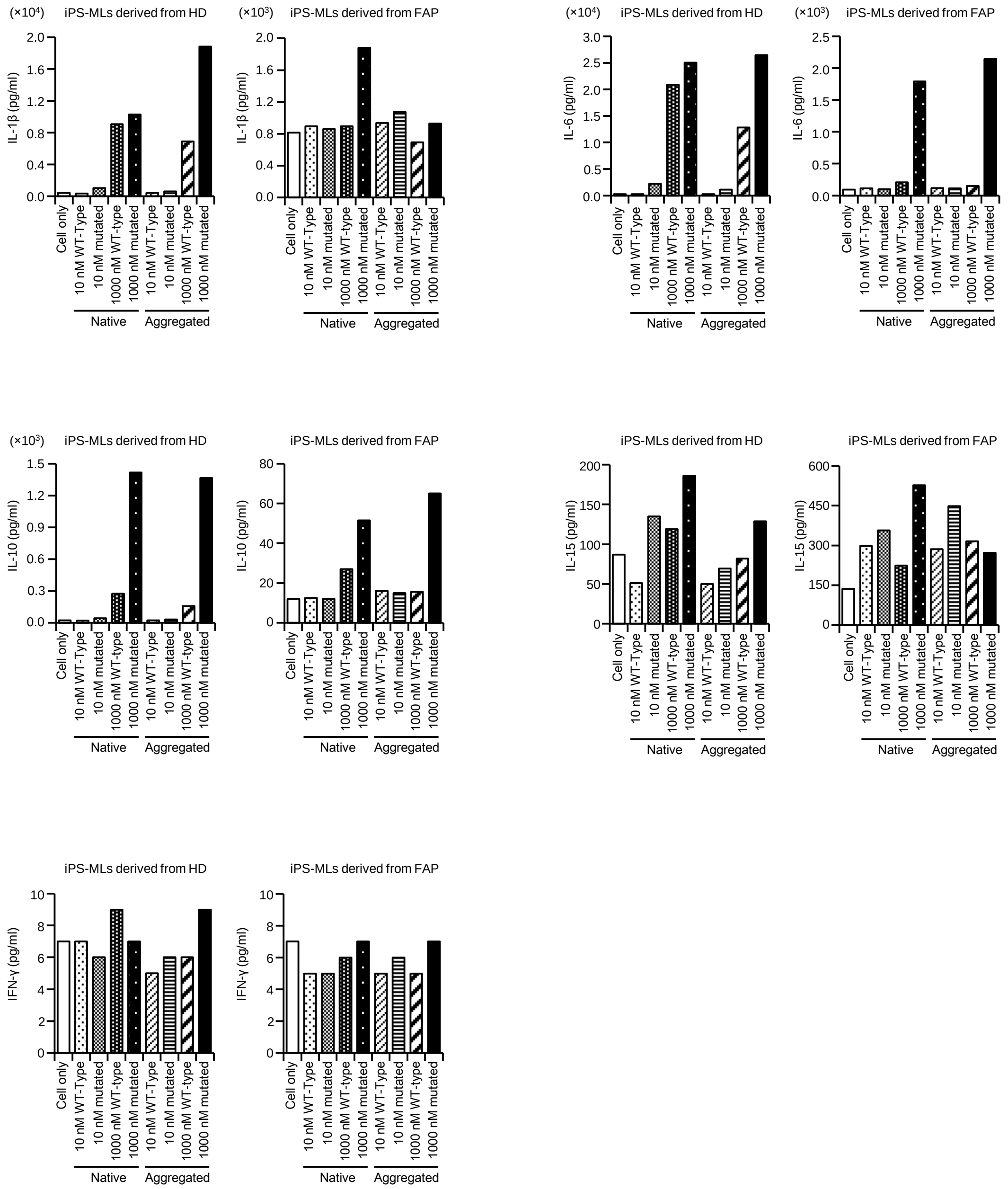

Supplementary Figure S2. Production of cytokines by TTR in HD and FAP-derived iPS-MLs. As described in the legend for Figure 2, healthy donor (HD) or familial amyloid polyneuropathy (FAP)-derived induced pluripotent stem cell-derived myeloid lineage cells (iPS-MLs) were cultured with each type of transthyretin (TTR) for 2 days. The Bio-Plex system was used to examine interleukin (IL)-1β, IL-6, IL-10, IL-15, and interferon (IFN)-γ concentration in culture supernatants.

Supplementary Figure 3 IL-10

(a)

CD4⁺ T cell

CD8⁺ T cell

CD14⁺ monocyte

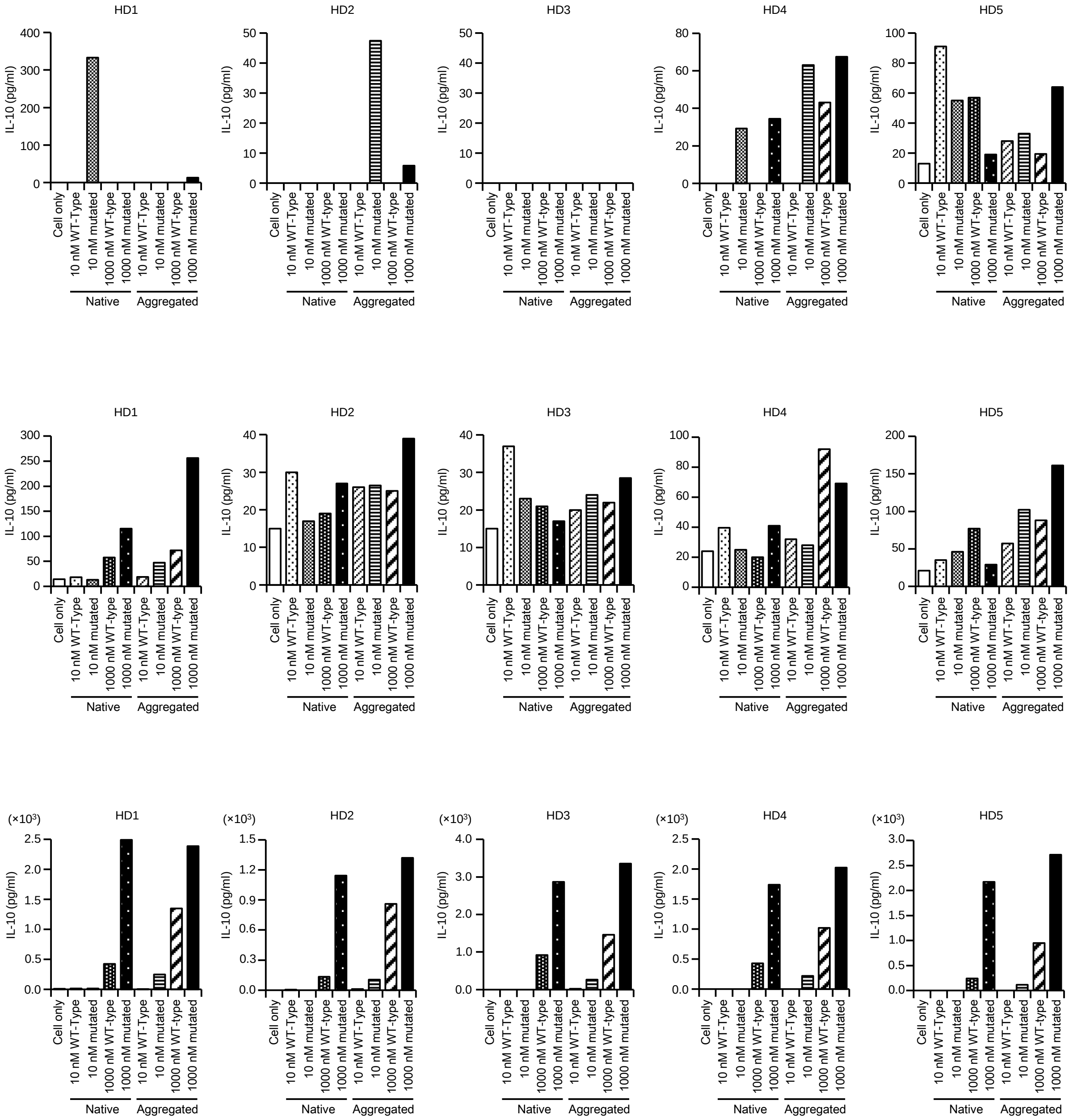

Supplementary Figure 3 IL-15

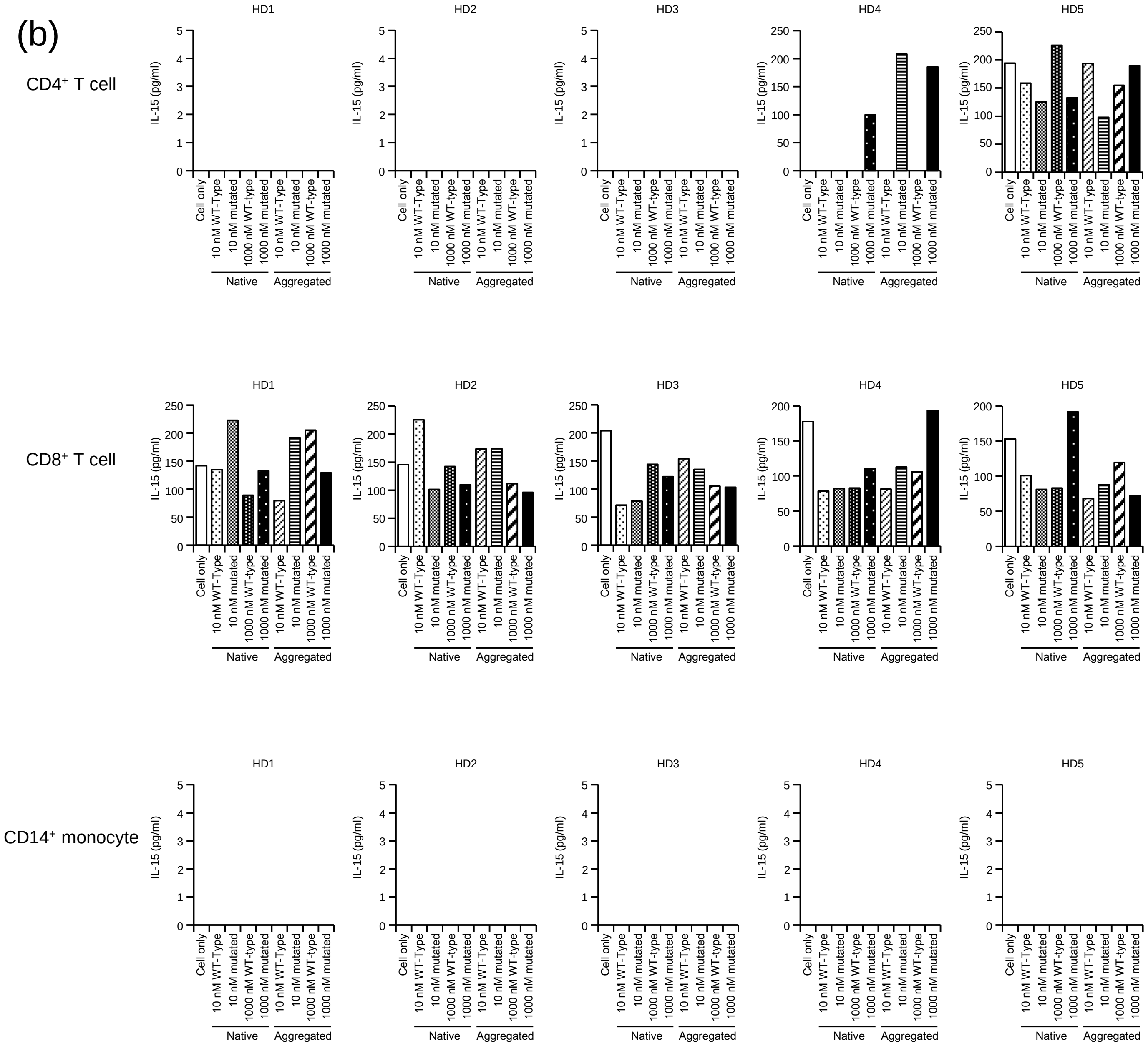

Supplementary Figure 3 IFN-γ

(c)

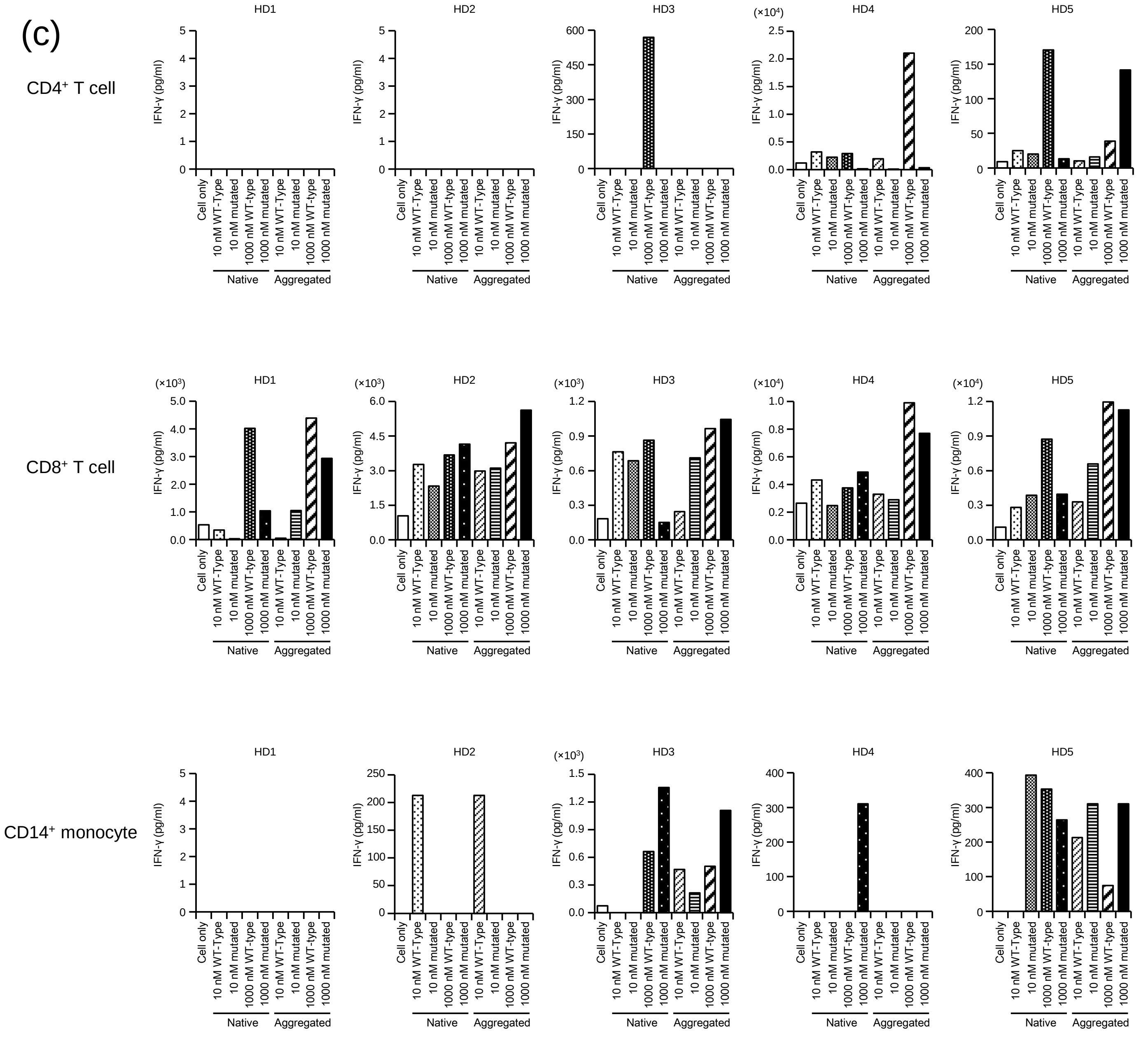

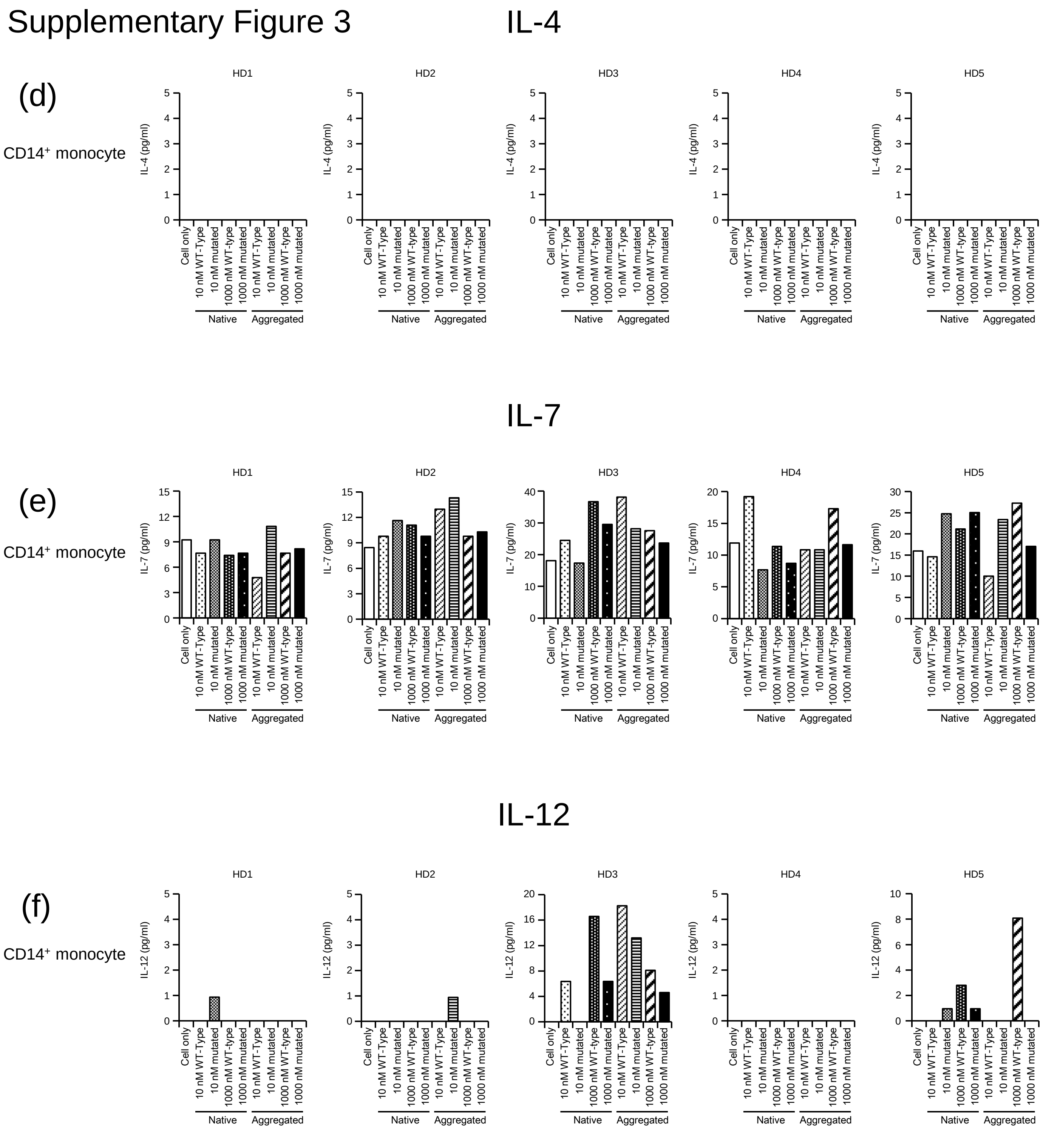

Supplementary Figure S3. Production of cytokines by transthyretin. As described in the legend for Figure 2, concentration of interleukin (IL)-10 (a), IL-15 (b), and interferon (IFN)-γ (c) was examined in culture supernatants of CD4+ T cells, CD8+ T cells, and CD14+ monocytes. IL-4 (d), IL-7 (e), and IL-12 (f) in the culture supernatant of CD14+ monocytes were also analysed.

Supplementary Figure 4

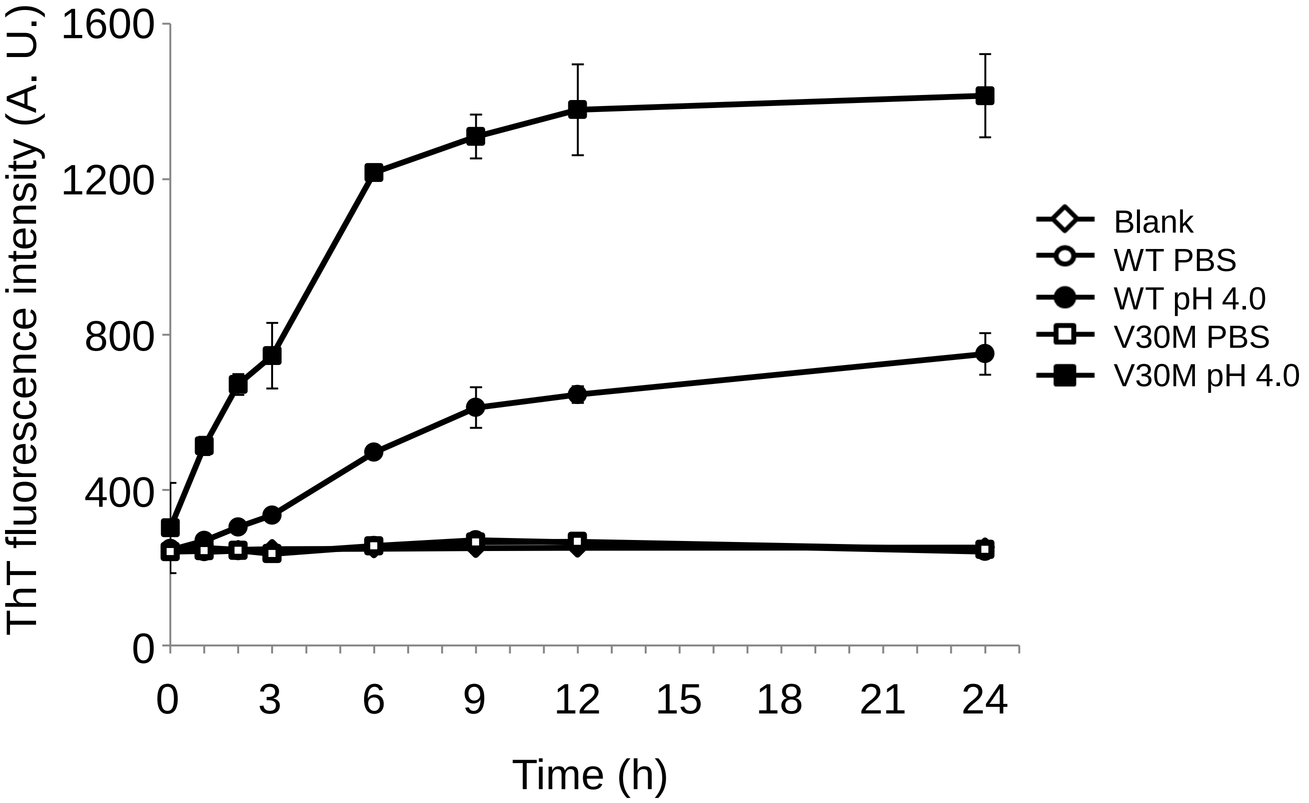

Supplementary Figure S4. Assessment of aggregated wild-type or mutated transthyretin.

Samples of wild-type or mutated transthyretin (TTR) (100 μM, pH 4.0) were incubated at 37°C for 24 h. For thioflavin T (ThT)-based fluorimetric assay, samples were mixed with ThT reaction buffer and ThT fluorescence intensity measured.

Supplementary Figure 5

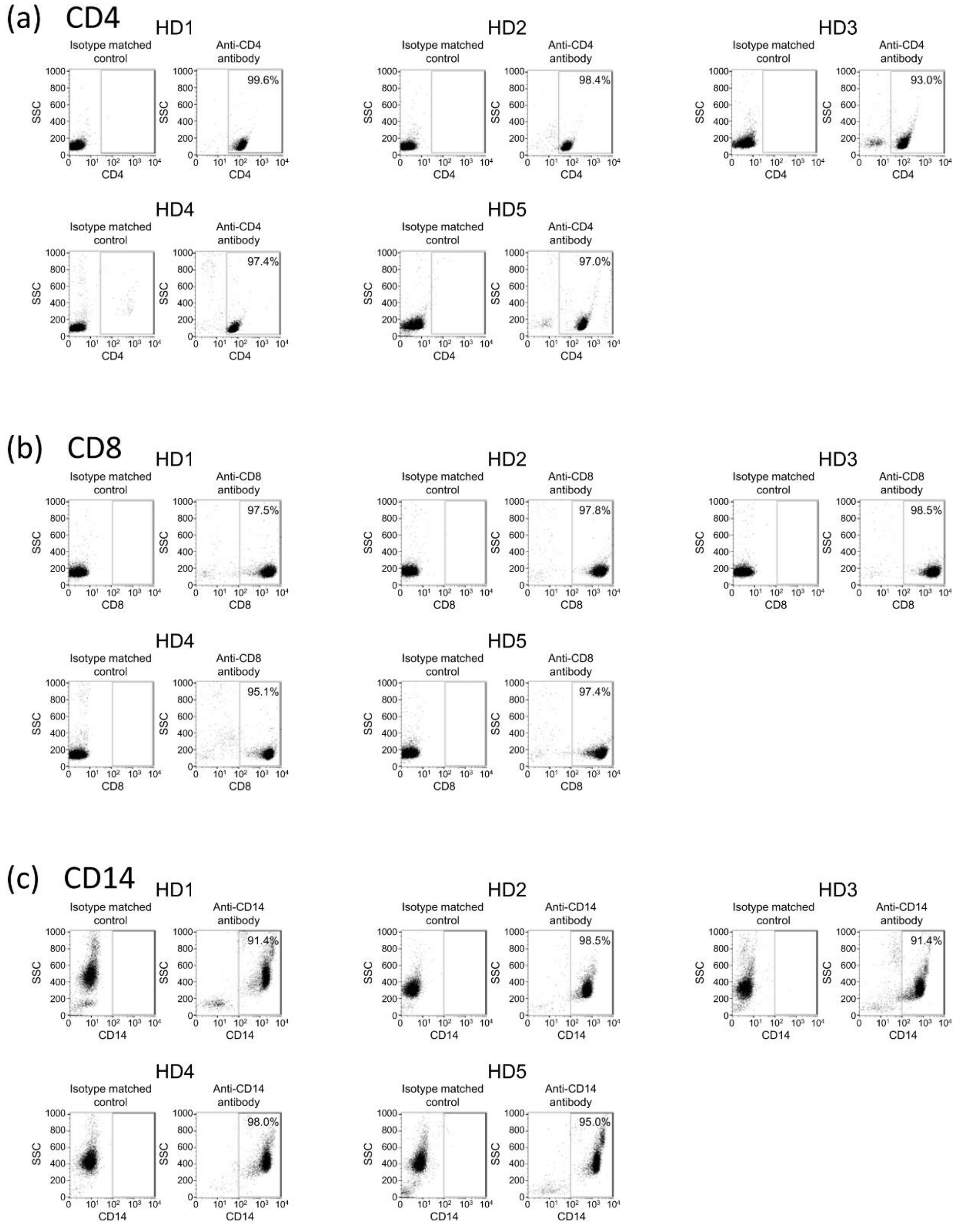

Supplementary Figure S5. Cell purity in CD4+ T cells, CD8+ T cells, and CD14+ monocytes. The proportion of CD4+ T cells (a), CD8+ T cells (b), and CD14+ monocytes (c) was verified by flow cytometry using a magnetic-activated cell sorting (MACS) system, with the results represented by dot plots.
